# The Relationship Between Low Serum Vitamin D Level and Early Dental Implant Failure: A Systematic Review

**DOI:** 10.7759/cureus.21264

**Published:** 2022-01-15

**Authors:** Lujain Alsulaimani, Abdullah Alqarni, Ammar Almarghlani, Mawadah Hassoubah

**Affiliations:** 1 General Dentistry, Dentistry Program, Ibn Sina National College for Medical Studies, Jeddah, SAU; 2 General Dentistry, Faculty of Dentistry, King Abdulaziz University, Jeddah, SAU; 3 Periodontics, Faculty of Dentistry, King Abdulaziz University, Jeddah, SAU; 4 General Dentistry, Dental Hospital, Ministry of Health, Yanbu, SAU

**Keywords:** low serum level of vitamin d, vitamin d supplements, vitamin d deficiency, dental implants, implant failure

## Abstract

The variety in shape and type of dental implants in the present time is considered one of the most successful evolutions in dentistry. This facilitates dental treatment options to restore patient function and appearance. However, numerous significant factors influence the predictability of survival or the success rates of dental implants, some of which, such as vitamin D levels, have not been included in many studies. The main purpose of this systematic review was to investigate whether there is a relationship between low serum levels of vitamin D and early dental implant failures (EDIFs). Our literature search involved international databases including PubMed, Directory of Open Access Journals (DOAJ), and Web of Science. Initially, according to our search criteria, 1200 studies were found. After excluding duplicates, incomplete studies, and studies not meeting our inclusion criteria, only six human studies were included in this research and analyzed. Finally, upon meticulous analysis of included studies, this systematic review revealed inconsistent results in articles with respect to the association between vitamin D deficiency and implant failures. Large-scale studies, especially clinically relevant studies, on this subject is recommended.

## Introduction and background

Because of their high survival rates, dental implants have shown to be a viable therapy for replacing missing and extracted teeth [1,2]. Implant bone integration (osseointegration) is essential during the early healing phase in order to obtain a clinical fixation under functional load that can be maintained over time [3,4]. Many factors can influence osseointegration, including surgical, prosthetic, implant, and patient-related factors such as the surgery technique, skills, and experience, timing and type of prosthetic loading, type of material used in prosthetic rehabilitation, design, and the surface of the implant, quality and amount of bone at the recipient site, postoperative inflammation or infection, smoking habits, and the immunological and nutritional status of the body [2,5,6,7,8,9].

A dental implant failure can be classified as "early dental implant failure" (EDIF) or "late dental implant failure" (LDIF) based on chronological criteria. EDIF is caused by a lack of osseointegration and indicates inadequate bone healing; on the other hand, LDIF is caused by the breakdown of osseointegration over time [10,11]. EDIF, which happens in a specific group of patients (systemic health condition), is one of the most researched problems in modern implantology and is difficult to manage [11,12,13].

Recognizing systemic risk factors may reduce implant failure and enhance predictability [12,13,14,15]. Some factors, such as a lack of vitamin D, may have a role in the emergence of EDIFs. In its inactive form (vitamin D3 or cholecalciferol), vitamin D is a steroid hormone that may be gained from diet or generated in the skin from cholesterol with adequate sun (ultraviolet light) exposure [16]. Vitamin D deficiency has long been known to impair the appropriate immune response to oral microbial infections and increase the risk of periodontitis [17]. Furthermore, vitamin D is required for bone metabolism and might impair healing and new bone formation on the implant surface [18]. Vitamin D stimulates osteoclast activity and the production of extracellular matrix proteins by osteoblasts. Currently, a serum 25(OH) level of 10 ng/mL is considered inadequate, while a level of 10-30 ng/mL is considered insufficient. The optimum serum level is more than 30 ng/mL [18,19,20,21].

Vitamin D deficiency is a global public health concern that can be caused by a combination of low food intake and insufficient sunlight exposure, obesity, and advanced age [22,23]. According to a recent Saudi Arabian study, women are four times more likely than males to have vitamin D insufficiency [24]. In addition, citizens in the eastern region are three times more at risk of developing vitamin D deficiency than those in other regions [25]. Recent review studies advocated for more research on vitamin D insufficiency in the context of dental implant failures [21,22,23]. More emphasis is being placed on encouraging the consumption of micronutrients, which may have health benefits and enhance resistance to diseases [26]. Furthermore, a certain diet and micronutrient may play a significant role in the different stages of dental implant healing and influence bone metabolism. A recent article, for example, has shown that there are direct impacts on the jaw and alveolar bone [27].

However, only a few well-designed investigations have looked into the relationship between vitamin D levels and EDIF. The majority were conducted on animal models, with only a few on humans [28,29,30,31]. The goals of this review were to evaluate the role played by vitamin D in implant survival rate, research the effects of low vitamin D levels on osseointegration, and determine the amount of vitamin D that can affect the implant survival rate.

## Review

Materials and methods

Study Protocol and Research Question

Preferred Reporting Items for Systematic Reviews and Meta-Analyses (PRISMA) criteria were used to perform a systematic review of research on the relationship between low serum vitamin D levels and EDIF [32]. The present systematic review investigated the following research question: Compared to a normal level of vitamin D, will a low level increase the risk of EDIF? The Population, Intervention, Control, Outcome (PICO) elements were identified as shown in Table 1.

**Table 1:** PICO elements PICO: Population, Exposure, Control, Outcome

Population	Completely or partially edentulous human adults restored with implant‐supported prostheses.
Exposure	Patients with low serum vitamin D level.
Control	Patients with normal serum vitamin D level.
Outcome	Dental implant failure (primary outcome), peri-implant marginal bone loss (secondary outcome), and biological (i.e., peri‐implant mucositis or peri-implantitis) or mechanical complications reported at the implant or patient-level (secondary outcome).

Selection Criteria

Inclusion criteria: The articles had to be in-vivo studies of male-female human adult patients ranging in age from 18 to 65 years old, written in English with full-text access, contain vitamin D-deficient individuals who received at least a single dental implant, and presence of hypothyroidism confirmed by laboratory testing (thyroid panel-TSH, free T4, free T3, or total T3) or medication. Papers were restricted to randomized controlled trials, case series, case reports, or prospective/retrospective studies. Finally, no time constraints were imposed in order to include as many studies as feasible in this systematic review (Table 2).

**Table 2:** Inclusion and exclusion criteria EDIF: early dental implant failure

Inclusion Criteria	Exclusion Criteria
Human adult patients (male-female) ranging in age from 18 to 65 years.	In-vitro and animal studies.
Studies written in English with full-text available.	Studies written in languages other than English.
Studies including vitamin D deficient patients who received at least one dental implant.	Wrong comparator = no comparison between vitamin D and EDIF.
Randomized controlled trials, case series, case reports, and prospective and retrospective studies.	Irrelevant outcome = outcome was unrelated to low vitamin D/implant failure.
Presence of hypothyroidism confirmed by laboratory testing (thyroid panel - TSH, free T4, free T3, or total T3) or medication.	Journals of publication not cited in the open-access checklist for predatory publishers.
No restrictions were placed on year of publication.	

Exclusion criteria: Studies excluded were those published in any language other than English, in-vitro studies (on animals), studies that lacked comparison or represented an inaccurate comparison between vitamin D and EDIF, studies published in journals not cited in the open-access checklist for predatory publishers, and/or with an irrelevant outcome in relation to low vitamin D/implant failure (Table 2).

Data were gathered using the terms "vitamin D," "vitamin D deficiency," "dental implants," and "implant failure" in international databases such as PubMed, Directory of Open Access Journals (DOAJ), and Web of Science. The study types identified were two case reports, two retrospective studies, one systematic search of peer-reviewed articles, and one scoping review. Data were collected according to different variables, such as year, location, the study’s goal/methodology, and results. They were divided in order by the authors’ name, year of publication, country, type of study, and the patients’ sample size, age, and sex. A meta-analysis was not conducted in this study because of the inconsistency in the results.

Results

**Figure 1:**
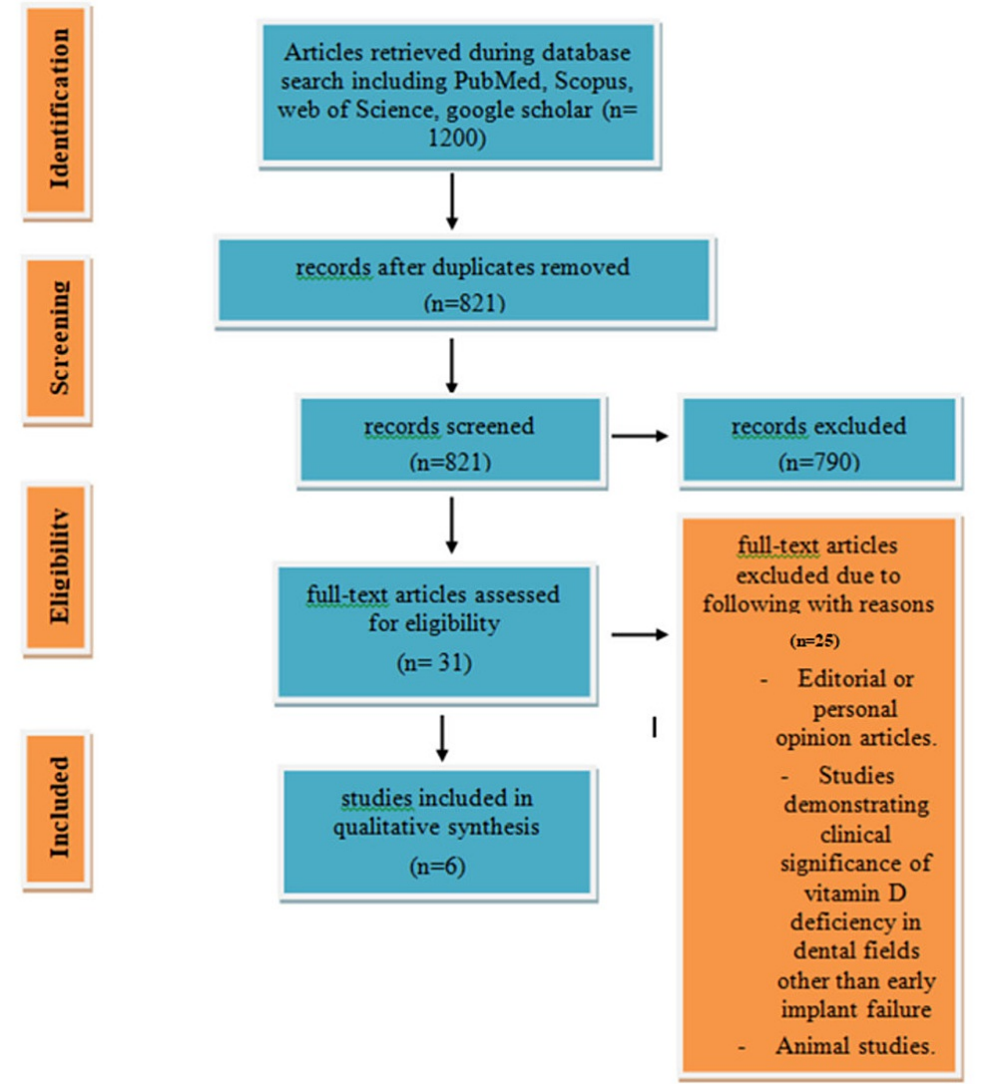
Flowchart describing the study design process

In the initial search, 1200 studies were found. After unrelated, duplicated, and incomplete information was eliminated, 31 studies were retained for the review. Of these, 25 were excluded because of not meeting the inclusion criteria. Finally, only six studies remained for review. The steps and criteria in selecting studies are given in Figure 1. The essential data of the six articles, including the author’s name, year of publication, location, design/type of study, sample size, and study results are displayed in Table 3. The studies selected were conducted in three different countries: Germany, Italy, and France. The largest number of studies, three, was conducted in Italy. This review includes six human studies (in vivo). Of these, two studies found insignificant relation between deficiency of vitamin D and dental implants failure, while the others reported a significant association between the two factors. The studies were evaluated, and any bias they included is reported.

**Table 3:** Summary and details of the included reports EDIF: early dental implant failure

Title of the article	Author	Country	Year	Design/type of study	Sample size	Results
1. Two neglected biological risk factors in bone grafting and implantology: High low-density lipoprotein cholesterol and low serum vitamin D.	Choukroun et al. [21]	France	2014	A systematic search of peer-reviewed literature.	________	Vitamin D serum level needs to be explored systematically in patients who are diabetic, allergic, hypertensive, and with a previous difficult case of implants and/or bone grafting.
2- Is low serum vitamin D associated with early dental implant failure? A retrospective evaluation on 1625 implants placed in 822 patients.	Mangano et al. [31]	Italy	2016	A retrospective clinical study (case-control study).	822 patients treated with 1625 implants	There were 27 EDIFs noted. There was no relationship found between gender, age, smoking, or a history of periodontitis and a greater prevalence of early failures. There were nine EDIFs in patients with blood vitamin D levels greater than 30 ng/mL, 16 EDIFs in patients with levels between 10 and 30 ng/mL, and two EDIFs in patients with low vitamin D levels.
3- Vitamin D deficiency as a suspected causative factor in the failure of an immediately placed dental implant.	Bryce and MacBeth [33]		2014	A case report	One 29-year-old patient	Five months postoperatively, no osseointegration around the dental implant was noticed. Medical tests showed that he was significantly vitamin D deficient, which may have contributed to the implant failure. Prior to implant implantation, individuals who have been in long-term hospital care or rehabilitation should have their vitamin D levels evaluated.
4- Low serum vitamin D and early dental implant failure: Is there a connection? A retrospective clinical study on 1740 implants placed in 885 patients.	Mangano et al. [34].	Italy	2018	Original Article: A retrospective clinical study (case control study).	885 patients treated with 1,740 fixtures	In all, 35 EDIFs were reported. There was no link discovered between EDIF and the patients' gender, age, smoking habits, or history of periodontal disease. In 27 individuals with vitamin D serum levels of 30 ng/mL, three EDIFs were found. Within its limitations (retrospective design, limited number of patients with poor vitamin D blood levels recruited), this study failed to show a meaningful relationship between low serum vitamin D levels and an elevated risk of EDIF. However, there was a strong tendency toward an increased incidence of EDIF with lower serum vitamin D levels, although further research is needed to better understand the link. There was no substantial link discovered between implant failure and vitamin D deficiency.
5. Vitamin D deficiency in early implant failure: Two case reports.	Fretwurst et al. [35].	Germany (Freburg)	2016	Case report	Two male patients (48 and 51 years of age).	All implants were implanted in two stages, and all had to be withdrawn within 15 days following implant placement due to vitamin D shortage (serum vitamin D level <20 μg/l). Both patients' implant placements were successful after they took vitamin D pills. To validate the relationship, more prospective, randomized clinical studies must be conducted.
6. Do dietary supplements and nutraceuticals have effects on dental implant osseointegration? A scoping review.	Nastri et al. [36].	Italy	2020	Scoping review	_______	This study reveals that nutraceuticals have a limited effect in promoting the osseointegration of dental implants. For example, there is a strong correlation between vitamin D shortage, poor osseointegration, and EDIF, necessitating proper supplementation.

Discussion

Few articles in the literature have investigated the association between osseointegration and low levels of serum vitamin D; the majority of them are animal experimental studies, while some are clinical research studies done on people. The purpose of this systematic review was to see if there was a relationship between vitamin D deficiency and EDIF. This review looked at six reports.

Despite the high success rate of dental implants, failure of dental implants has also been observed in certain circumstances. In Iran, Mohajerani et al. observed that 73 cases (6.68 %) of the 1,093 implants assessed failed in the early phases [32]. According to a study by Jafarian et al. in Iran, 61 (4%) of 1533 dental implants in 250 patients failed; the maxilla was found to have the greatest fracture rate (9 of 132 implants (6.8 %)) [37].

There are many reasons for dental implant failure, including failure of the bone to heal around the implant and consequent failure of osseointegration, infection, smoking, and a narrow, keratinized gingiva [38], as well as vitamin D insufficiency. Recognizing risk factors might minimize failure rates and improve the predictability of dental implant treatment. Four of the six articles reviewed revealed a relationship between vitamin D insufficiency and dental implant failures, while the other two found no relation.

Bryce and colleagues investigated the link between vitamin D insufficiency and rapid dental implant insertion. According to their case report, the patient was significantly vitamin D deficient, which may have jeopardized the implant's effectiveness [33]. Another research [31] looked at the relationship between low blood vitamin D levels and EDIF in 822 participants. The authors discovered nine EDIFs in participants with blood vitamin D levels greater than 30 ng/mL, 16 EDIFs in respondents with levels between 10 and 30 ng/mL, and two EDIFs in subjects with levels less than 10 ng/mL [31]. As a result, while no statistically significant link was discovered between serum vitamin D levels and EDIF, we did find an increase in early failures related to serum vitamin D levels. The study's approach had various benefits, including the restriction of the research to "early implant failures" [31].

A retrospective study with 885 patients expanded on the prior study [34] and appeared to validate the data that had previously emerged. In individuals with sufficiently high serum vitamin D levels (>30 ng/mL), the failure rate was modest. Early failures almost doubled in individuals with inadequate blood vitamin D levels (10-30 ng/mL) and were approximately four times higher in those with severe vitamin D insufficiency (10 ng/mL). As a result, the study found a link between an increase in EDIFs and vitamin D deficiency in the blood [34]. Given the rising frequency of vitamin D insufficiency worldwide [39,40,41], we need more studies researching the links between vitamin D and EDIF.

There are other reports, besides the ones included by us in our review, that look into the relationship between vitamin D and factors that may cause EDIF. Several micronutrients (vitamin D, magnesium, resveratrol, and vitamin C) were thought to influence the skeletal system, especially jaw bone and alveolar bone, as well as dental implant osseointegration [42]. In addition, Wagner et al. demonstrated that osteoporosis has a significant detrimental influence on marginal bone loss surrounding implants and that vitamin D treatment counteracts such loss, with overall positive effects on peri-implant bone growth [43]. During osteointegration, calcitriol impacts the activation and differentiation of osteoblasts and osteoclasts. Vitamin D also increases bone mineralization [44]. Furthermore, vitamin D is essential for immunity and the inflammatory response, allergies, as well as increasing anti-inflammatory and decreasing pro-inflammatory cytokines [45]. Bashutski et al. revealed that a low level of vitamin D in the serum reduced the advantages of periodontal surgery and hampered post-surgical recovery. Vitamin D has also been found to be an important part in the maturation and appropriate function of bone cells since it creates a factor that drives osteoclast precursor fusion and osteoblast differentiation [46].

The limitation of this review is the small number of clinical trials with large sample sizes. Our search revealed that there is a low number of clinical studies in this sector, and It is recommended that more large-scale studies with big sample numbers be carried out in the future to discover the specific relationship between vitamin D and EDIF.

## Conclusions

There are several causes of dental implants failure. Improper bone healing around the implant, smoking, infection, low keratinized gingiva are the most common reasons for early implants loss. According to the findings of this study, it is difficult to find a direct relation or causality between the low serum vitamin D level and EDIF. However, the serum level of vitamin D may play an important role in osseointegration and dental implant success or predictability of dental implant survival rate through its effects in modulating the immune system and healing process. More large-scale clinical and prospective studies need to be conducted on this hypothesis.
